# External Ventricular Drain (EVD) Placement Using a Hands-On Training Session on a Simple Three-Dimensional (3D) Model

**DOI:** 10.7759/cureus.28014

**Published:** 2022-08-14

**Authors:** Stacey Podkovik, Tye Patchana, Saman Farr, James Brazdzionis, Max Marino, Paras Savla, Samir Kashyap, Brian Chin, Andrew Crouch, Dan E Miulli

**Affiliations:** 1 Department of Neurological Surgery, Riverside University Health System Medical Center, Riverside, USA; 2 Department of Neurological Surgery, Riverside University Health System Medical Center, Moreno Valley, USA; 3 Department of Emergency Medicine, Arrowhead Regional Medical Center, Colton, USA; 4 Department of Neurological Surgery, Arrowhead Regional Medical Center, Colton, USA

**Keywords:** 3d printing, 3 dimensional printing, model, medical education, external ventricular drain

## Abstract

Neurosurgery is a demanding field with small margins of error within the operative field. Small errors can yield devastating consequences. Simulation has been proposed as a methodology for improving surgical skills within the neurosurgical realm. This study was conducted to investigate a novel realistic design for a clinical simulation based, low-cost alternative of external ventricular drain (EVD) placement, an essential basic neurosurgical procedure that is necessary for clinicians to master. A low-cost three-dimensional (3D) printed head using thermoplastic polylactic acid was designed with the tactile feedback of outer table, cancellous bone, and inner tables for drilling with replaceable frontal bones pieces for multi-use purposes. An agar gel filled with water was designed to simulate tactile passage through the cortex and into the ventricles. Neurosurgical and emergency resident physicians participated in a didactic session and then attempted placement of an EVD using the model to gauge the simulated model for accuracy and realism. Positioning, procedural time, and realism was evaluated. Improvements in procedural time and positioning were identified for both neurosurgical and emergency medicine (EM) residents. Catheter placement was within ideal position for all participants by the third attempt. All residents stated they felt more comfortable with placement with subsequent attempts. Neurosurgical residents subjectively noted similarities in tactile feedback during drilling compared to in-vivo. A low-cost realistic 3D printed model simulating basic neurosurgical procedures demonstrated improved procedural times and precision with neurosurgical and EM residents. Further, similarities between in-vivo tactile feedback and the low-cost simulation technology was noted. This low cost-model may be used as an adjunct for teaching to promote early procedural competency in neurosurgical techniques to promote learning without predisposition to patient morbidity.

## Introduction

Neurosurgery is a particularly unforgiving field of medicine in which even small operative errors have the potential to change the fundamental personality of a patient that makes them who they are. Throughout the nineteenth century, most medical training, particularly within surgery, has operated under the adage “see one, do one, teach one” formally implemented by Dr. William Halsted [1,2]. Given the high-stakes and unforgiving nature of neurosurgical procedures, greater attention must be placed on balancing resident learning and the principle of patient non-maleficence. While there is no perfect alternative for operating on a real patient, medical simulation has proven to be an effective method for applying newfound knowledge and technical skills in a controlled environment. In the eighteenth century, medical simulation existed mostly in the form of wax replicas [3]. In the 1950s, the first low fidelity mannequin, the Resusci-Anne, was created by Asmund Laerdal for cardiopulmonary resuscitation practice [3]. Since then, medical simulation has continued to evolve and has been utilized in a number of medical and surgical specialties.

The external ventricular drain (EVD) procedure is among the first, and most basic, procedures to master as a junior neurosurgical resident [4,5]. It involves placing a silicon-based catheter into the ventricular system to provide a diversion for cerebrospinal fluid (CSF). Although considered to be one of the most standard procedures within critically ill neurosurgical patients, it is a procedure that is typically done blindly at the bedside, relying on surface anatomical landmarks to help with trajectory planning and ideal catheter placement. Today, multiple different EVD training modalities exist, including cadavers, three-dimensional (3D) synthetic models and virtual reality (VR) simulators. As costs of medicine rise, there is an ever-mounting pressure to be well-versed in technical procedures so that a trainee is not “practicing on my loved one.” Herein, we present a 3D cast model with replaceable parts that offers trainees an accurate, safe, and reproducible form of EVD placement training with tactile feedback. Additionally, we provide the source code material necessary for training programs to reproduce this model for its own trainees.

## Technical report

This project was reviewed and approved by our institution’s local institutional review board. To help gauge the simulated model for accuracy and realism, a total of 11 resident physicians from Accreditation Council of Graduate Medical Education (ACGME)-accredited neurosurgery (n=5) and emergency medicine (EM; n=6) residency programs were recruited for this study. Residents were split into two groups, neurosurgery and EM, and underwent identical didactic training sessions followed by a series of practical hands-on evaluation sessions. Didactic sessions consisted of a PowerPoint presentation that was previously recorded in video format to maintain consistency across both groups. The three-minute training video introduced the procedure and reviewed the basics anatomical landmarks, as well as a brief step-by-step guide to performing the EVD procedure. Following the didactic sessions, both groups of residents used the 3D model to place of an EVD for a total of four attempts per individual. For each attempt, total time for procedure, accuracy of placement, number of attempts at passing the catheter needed during each procedure prior to successful placement of catheter (as indicated by obtaining CSF) and postgraduate year of training was noted. This same protocol was followed for both groups of residents.

Creating the model

**Figure 1:**
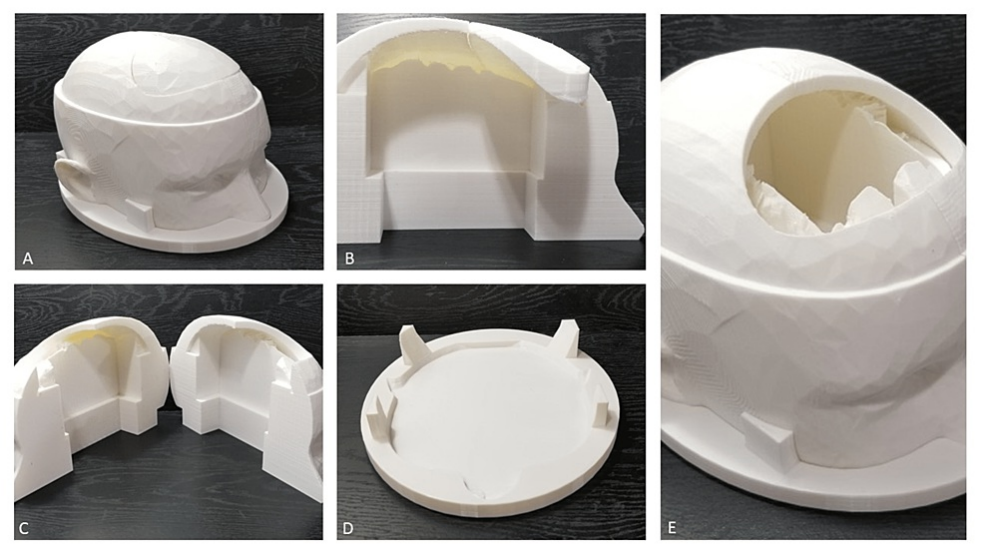
3D model of skull A) entire model, B) closed model side profile view, C) both halves of model opened, D) model base, E) replaceable frontal bone

**Figure 2:**
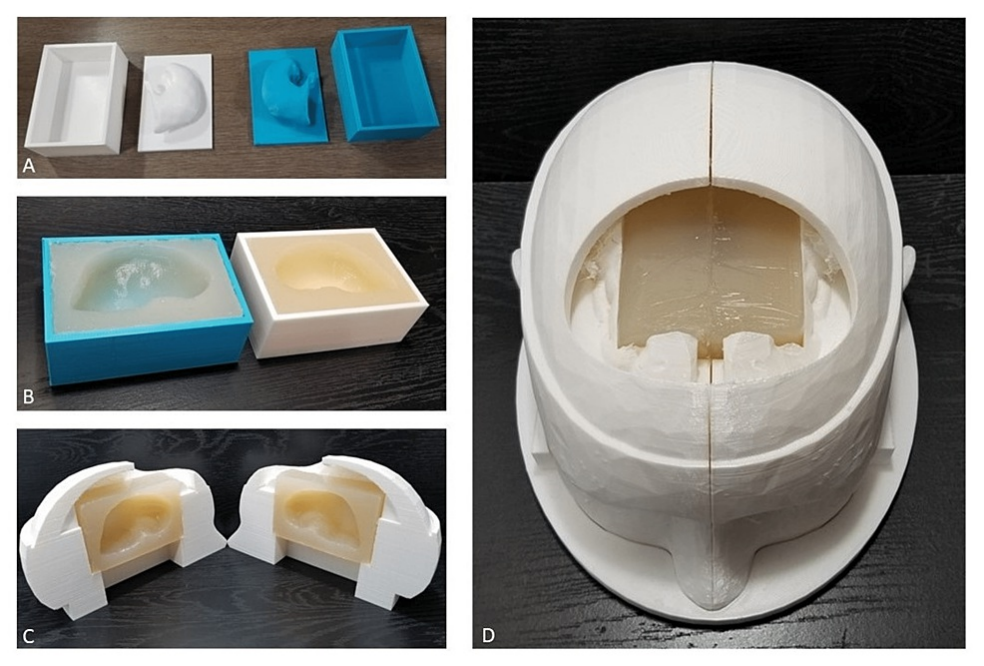
Model of skull with agar gel mold A) ventricular mold casts, B) ventricular cast with agar gel, C) agar gel in model, D) ventricular agar gel in entire closed model

The 3D head model was created using a free online computer-aided design (CAD) software, TinkerCAD (Autodesk, San Francisco, USA) and a thermoplastic polylactic acid (PLA) filament (Figure 1). The source code for the model has been taken from Burr Hole Placement (ARMC Simulation Labs, Aiken, USA). After initial design set up, the entire 3D printed model including the base and skull with 15 replaceable frontal bones can be made for $65 with the possibility of creating additional replacement skull pieces in sets of 10 for an additional $22. Print time is approximately 100 hours in summative time for this set up with approximately five hours needed post processing. The agar for the ventricular mold was created using “Premium Agar Powder for Baking and Cooking” created by PowderForTexture. The mold volume of each half of the ventricle is approximate 220 mL. The appropriate volume of water was heated to boiling temperature. A 3.5% agar solution by weight (3.5 g/100 mL) was then prepared by dissolving the appropriate weight of agar powder in the boiling water (depending on the number of molds to be made). Care was taken to add the agar powder very slowly while the solution was being stirred to prevent precipitation and clumping of the agar powder. Once all of the agar had dissolved, the solution was then cooled down to 50-60 degrees Celsius using a water bath. The solution was slowly stirred as it was cooled, to prevent solidification of the agar gel. After the solution was cooled, it was slowly poured into the ventricle molds (to avoid agitating the solution and creating air bubbles). The upper section of the mold was then placed on top to create the ventricle impression as the agar gel cools inside the mold. A small amount of agar solution was allowed to overflow out of the top of the mold as the upper portion was placed, to prevent any air pockets inside the mold. The mold was then allowed to cool down and set at room temperature for at least two hours before being used. If the agar gel was not intended to be used the same day, it was stored in a refrigerator in a closed container (to prevent desiccation) for up to four days prior to use. The agar mold can be quickly fit into the 3D cast model of the head (Figure 2). To create the tightest fit, the agar was wrapped in cellophane on all sides except the top to prevent any leakage of the CSF contents. The gel itself cost $30 for the entire experiment. Overall labor costs for 3D printing and gel creation are estimated at 15 hours for design time, with approximately 100 hours of print time. Overall person hours for quality evaluation of each printed skull, agar gel creation, and assembly is estimated to be six for approximately 10 complete skull models.

Using the model

**Figure 3:**
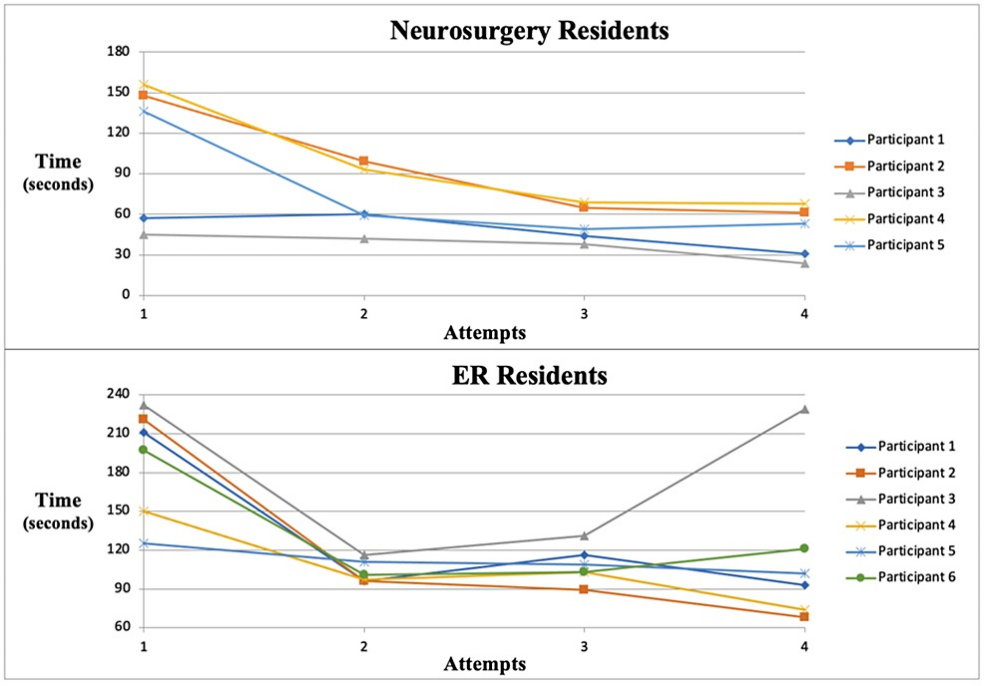
Line graph demonstrating time to properly place EVD with subsequent attempts for each participant EVD: External ventricular drain

There was an improvement in procedural time and precision of placement by residents in both groups corresponding to the number of attempts (Figure 3). One EM resident that had an aberrant fourth attempt due to improperly setting up the equipment. Detailed examination of catheter placement showed ideal trajectories for all participants by the third attempt. Senior neurosurgery residents had ideal positioning with every attempt, had fewer catheter passes per attempt than other participants, and saw a slight improvement in their placement times with subsequent attempts. All of the junior neurosurgery and EM residents stated that their comfort level improved with each subsequent attempt. Lastly, a Likert survey was sent out asking the residents if they found the model useful to their training, with a 100% response rate stating they strongly agree that this model was useful for their training and was a realistic simulation for the procedure.

## Discussion

The current landscape of neurosurgery requires that a trainee develop a great deal of precision, accuracy, and knowledge well before they ever pick up a scalpel. The EVD is most commonly one of the first procedures that junior residents learn, and then are given the ability to perform on their own without direct supervision [4,5]. Despite being one of the first invasive procedures learned, it presents with its own difficulties: it is a blind procedure without navigation, and the surgeon must rely on external landmarks and tactile feedback to approximate the ideal catheter trajectory and position. Complications of catheter misplacement can include worsening hydrocephalus, hemorrhage, infections, malfunctioning drains, need to replace the drain, or injury to surrounding cortical tissue [4,6]. These types of sequelae can lead to catastrophic outcomes, and trainees need to have confidence in both doing the procedure and complication management.

The conventional “see one, do one, teach one” method of teaching has slowly been replaced with “see one, simulate one, do one” [1,2]. Due to the presence of higher patient safety standards, and work hour restrictions, resident physicians have encountered many obstacles in terms of intra-operative practice as compared to previous generations [1,3]. In order to accommodate for these hurdles, the need for newer training modalities for young neurosurgeons has sparked a great deal of ingenuity in the field. Governing bodies in charge of resident education have begun requiring simulation based training models to be incorporated within the curriculum for other surgical specialties such as general surgery. Unfortunately, neurosurgical advancements regarding simulators has been lacking as compared to other specialties [5].

Currently, aside from cadaveric models, there have been multiple simulators such as ImmersiveTouch (ImmersiveTouch Inc., Chicago, USA), SIMONT (ProDelphus, Brazil), NeuroSIM (Physical Optics Corporation, Torrance, USA), MARTYN (Royal College of Surgeons England, England), BabyMARTYN (Royal College of Surgeons England, England), and other VR devices that have shown promising results in terms or providing resident surgeons with early procedural training that can be reproduced in the operating room [1,2,6-9] All simulators have the goal to improve procedural knowledge [10].

Cadaveric models are challenging because of the high costs associated with obtaining and maintaining them, compounded with the fact that the tissues and morphologies are not very representative of in-vivo procedures [2,4,10,11]. Byvaltsev et al. described the use of 3D printed models over animal models. However, this comes at the cost of not replicating true human anatomy [12]. VR simulators have the benefit of excellent anatomic representation. However, these simulators come at a very high cost, ranging from $6,000 to $80,000, with additional maintenance costs [3,5,8] and do not provide the same tactile feedback. Physical simulators appear to provide better procedural preparation and execution, allowing surgeons to use surface landmarks and tactile feedback to help plan appropriate trajectories as well as developing a sense of patient positioning and hand and instrument placement [1-3,8,10,12]. The new ability to use 3D printers allows for cheap production of the models, ranging between $4 and $20 [4,10].

A method to assess the model, to gauge the simulated model for accuracy and realism, and to teach residents a procedure was developed. The residents in both cohorts demonstrated improvements in their times and precise placement. Although the neurosurgical residents showed faster times during all their attempts as compared to the EM residents (which is to be expected as that cohort consisted of mostly senior residents who had established techniques), they also demonstrated an improvement in time for the subsequent trials as well as placement precision. The EM residents did demonstrate improved procedure times as well as improved technique and placement positioning with subsequent attempts. The improvement in times within each cohort shows that regardless of training, each resident physician became increasingly familiar with the model and steps of the procedure, improving their efficiency. In the EM cohort, the improvement in catheter position demonstrates that physicians that are novices to a procedure receive benefit in their technical skills through the use of 3D realistic anatomic models. Each resident appreciated the tactile feedback which led to better precision in the cranial caudal direction.

Our model consists of a 3D printed cast made from a thermoplastic called American Board of Surgery (ABS) filament. We created a replaceable portion of the frontal bone so that residents could simulate drilling without having to dispose of the entire model afterwards. Breimer et al. found that certain physical simulators have a long shelf life [8], and the use of a replaceable piece can help the longevity of the model. The agar mold of the ventricular system comes in two halves, allowing not only for tactile feedback but also for the trainee to visualize where the ventricular catheter punctures and where the tip terminates. This provides the resident real-time feedback on how to make adjustments with their trajectory planning. With the ability to have a removable top that permits almost four separate initial twist drill holes, the resident would be able to make multiple attempts at the procedure with subtle refinements in their technique. Additional attempts would only require the replacement of the top, and possibly the ventricular mold, allowing for the reuse of the remainder of the model leading to decreased production costs. This aids in procedural reinforcement and is an invaluable self-teaching tool.

A large limitation of this study was the low number of participants that attempted ventricular placement of the model. However, the variety of residents and postgraduate year provided feedback about realism, tactile sensation, and the ability to make small adjustments for precision. In order to adequately assess specific levels of proficiency and statistically significant improvement in technique, a larger sample size would be needed to have sufficient statistical power to make conclusions. Additionally, based on resident availability at the time, it was mostly senior neurosurgery residents (years four and above) that participated in the cohort in addition to a wide array of EM residents. This meant that most of the residents within the neurosurgical cohort had already had practice with EVDs, and their times for their initial attempt were significantly faster than those in the EM cohort, thereby showing a smaller improvement in consecutive attempts.

## Conclusions

3D models and didactics demonstrate an effective strategy for resident physician procedural training. We created an inexpensive 3D model to be used for EVD placement and had positive feedback from two resident cohorts in terms of the realistic nature of the model and the improved comfort that they developed with subsequent catheter placement attempts. The simulation model presented allows for real-time tactile feedback, visualization of final ventricular catheter placement, and tip termination, as well as a method to train and improve a basic procedure for the novice and senior resident.
